# Knowledge and practice of community pharmacists regarding the safety of drugs during pregnancy: a cross-sectional study from a developing country

**DOI:** 10.1186/s12884-024-06393-3

**Published:** 2024-03-11

**Authors:** Amer A. Koni, Hamzah Qashoa, Abeer Abo Musa, Maen Masri, Walaa Hazem, Sari Taha, Aiman Daifallah, Samah W. Al-Jabi, Amani S Abushanab, Sa’ed H. Zyoud

**Affiliations:** 1 Department of Clinical and Community Pharmacy, Faculty of Medicine and Health Sciences, An-Najah National University, Nablus, 44839 Palestine; 2 Division of Clinical Pharmacy, Department of Hematology and Oncology, An-Najah National University Hospital, Nablus, 44839 Palestine; 3 Department of Medicine, Faculty of Medicine and Health Sciences, An-Najah National University, Nablus, 44839 Palestine; 4 Poison Control and Drug Information Center (PCDIC), Faculty of Medicine and Health Sciences, An- Najah National University, Nablus, 44839 Palestine; 5 Clinical Research Center, An-Najah National University Hospital, Nablus, 44839 Palestine

**Keywords:** Pregnancy, Community pharmacists, Drug use, Knowledge, Practice

## Abstract

**Background:**

Drug use during pregnancy can cause unfavorable fetal and maternal outcomes. Information sharing is essential for pharmacists’ role within intricate, modern healthcare systems. Community pharmacists (CPs) have demonstrated unsatisfactory knowledge across various pharmacological domains in most developing countries. This study aimed to explore the knowledge and practices of CPs regarding medications and herb safety during pregnancy.

**Methods:**

A cross-sectional study was conducted in a developing country using a self-administered questionnaire. A sample of CPs working in the northern governorates of the West Bank was selected by convenience sampling. The questionnaire included questions on sociodemographic characteristics, practices and knowledge. Descriptive and inferential statistics were calculated using the Statistical Package for the Social Sciences (SPSS) to analyze the data.

**Results:**

A total of 207 questionnaires were completed. Most respondents had only a bachelor’s degree (89.9%) but did not participate in continuous professional development (CPD) (71.0%). Almost one-third of the CP workload involved dispensing drugs to pregnant women. The majority of the participants reported that they inquire about pregnancy status (59.9%), refer to scientific sources (82.6%), and contact a prescribing physician (51.2%) in cases of uncertainty. A higher knowledge score was associated with receiving a master’s degree and CPD programs. Most CPs identified folic acid, paracetamol and amoxicillin as safe, while tetracycline, isotretinoin, enalapril, pseudoephedrine and ibuprofen were among the drugs mostly reported as unsafe. Castor oil, Senna, St. John’s wort and ginseng were the most frequently reported herbs as unsafe.

**Conclusions:**

Despite the gaps in knowledge about herb pharmacology, CPs demonstrated acceptable knowledge and practice regarding drug safety during pregnancy. CPD is recommended for addressing gaps in knowledge and practice. Future research evaluating knowledge and practice may benefit from developing a specific, accurate, validated instrument.

**Supplementary Information:**

The online version contains supplementary material available at 10.1186/s12884-024-06393-3.

## Background

Inappropriate drug use during pregnancy poses considerable risks to both the mother and the fetus, causing maternal complications and adverse fetal outcomes [1–4]. Assessment of potential fetal harm resulting from drugs is challenging due to ethical considerations [5]. Pregnancy-related data on most available drugs are derived from observational studies. In the premarketing stage, data on medication safety are based on experimental animal studies, as new drugs do not include pregnant women in clinical trials. However, the reliability of establishing a pregnancy safety profile at that stage is limited due to difficulties in extrapolating from these animal studies [6].

Medical regulatory authorities worldwide have developed risk classification systems that categorize medications based on the associated risks and benefits while recognizing the limitations of such classifications and the importance of case-by-case clinical evaluation [7, 8]. Indeed, evaluating drug safety is complex. The teratogenic risk of drugs, for example, depends on several factors, such as drug dosage, route of administration, treatment duration, and gestational age during which the drug is used [1, 9]. Moreover, physiological changes during pregnancy may alter the pharmacokinetic properties of drugs [10].

Drugs may be used during pregnancy to manage preexisting health conditions such as hypertension and diabetes; treat medical complications of pregnancy such as preeclampsia; and alleviate pregnancy-induced symptoms such as nausea and vomiting [11–14]. The most frequently used medications during pregnancy include antibiotics, antifungal agents, iron tablets, analgesics, antiemetics and antacids [15, 16]. This is in addition to self-medicating with over-the-counter (OTC) medications and herbs, which are more difficult to assess. While global estimates of drug use during pregnancy vary across different settings, most studies indicate a high utilization rate. A large web-based survey showed that more than 80% of pregnant women in the Americas, Australia and Europe used at least one medication, either prescribed or OTC [12]. Regionally, a study from Saudi Arabia reported that almost 40% of women used medicines or herbs during pregnancy [17]. In Palestine, the utilization of drugs during pregnancy is common. A study of pregnant women revealed that more than two-thirds of participants reported taking prescription-only medications, while most took vitamins, iron, and calcium supplements [18]. Another study investigated the use of complementary and alternative medicines (CAMs) among pregnant women. Most participants (87.7%) used nonherbal, biologically based drugs, including vitamins and supplements [19]. In addition, the use of herbs during pregnancy was found to be prevalent, ranging between 77.1% and 88.4% [20, 21].

In the modern medical environment, the role of pharmacists has expanded from the mere dispensation of accurate and safe drugs to the collaborative and specialized coordination of pharmaceutical care [22]. The services provided by pharmacy practice have diversified to include pharmaceutical counseling, patient education, medication therapy management, and collaboration with other healthcare professionals. Most importantly, pharmacists have become responsible for optimizing and transitioning patient care across different settings [23]. As the pharmacist is one of the most accessible healthcare professionals, information sharing is key to fulfilling the pharmacist’s duty and developing pharmacist-patient relationships [22, 24].

Although the main role of community pharmacists (CPs) in Palestine is still focused on dispensing medication with limited patient communication [25, 26], CPs seem to influence patients’ decisions. Two studies demonstrated the significant influence of CPs on patients’ decisions to self-medicate and use CAM [27]. This is especially relevant for antibiotics, which can be purchased without a prescription in Palestine [27]. However, pharmacists in Palestine had fair-to-average knowledge of several pharmacological topics, including side effects and contraindications for drugs, bioequivalence and pharmacokinetics, drug-food interactions, antibiotic resistance, and CAM [28–31]. This knowledge gap implies possible poor practices regarding the use of medications during pregnancy. To date, no study has explored the knowledge of CPs regarding the use of medications during pregnancy, which is key to informing educational and regulatory interventions to improve pharmacy practices. This study aimed to investigate the knowledge of CPs regarding the use of prescribed medications, OTC medications and herbs during pregnancy in Palestine.

## Methods

### Study design

This was a cross-sectional study based on a self-administered questionnaire (Additional file 1). All licensed community pharmacists working in the governorates of Nablus, Tulkarm, Qalqilya, and Salfit in the northern region of the West Bank were eligible for inclusion.

### Sampling

The Raosoft online sample size calculator was used to estimate the sample size (http://www.raosoft.com/samplesize. html). Based on information from the Palestine Pharmacists Syndicate, 1856 licensed community pharmacists worked in the northern governorates of the West Bank. With a predetermined margin of error of 5% and a confidence level of 90%, the minimum sample size was 237. To account for potential nonresponse and missing data, the surveyed sample size was increased to 250. A convenience sampling technique was employed because obtaining a comprehensive list of pharmacists and their workplace locations is impractical and infeasible.

### Questionnaire

The initial form of the questionnaire was constructed based on previous studies [28–31] and cross-reviewed for content validity by a group of professors and pharmacists with experience in clinical pharmacy, biostatistics and research. In this cross-review process, the medication list was developed independently by each expert, cross-checked, and edited to include the most common medications, supplements, and herbs used locally. The selection of medications ensured the development of a comprehensive list of the various types and indications of medications, such as antibiotics, bronchodilators, oral contraceptives, and pain killers.

The questionnaire was forward-translated to Arabic, the native language of the respondents, by two independent translators to address discrepancies between the translations. The questionnaire was subsequently back-translated to English to ensure the accuracy of the original translation. To test the feasibility and acceptability of the questionnaire and ensure its face validity, a pilot study was conducted on 20 respondents who were excluded from participating in the primary study. Guided by the opinions of the respondents in the pilot study, minor inaccuracies in language and content were improved. The time needed to complete the questionnaire was approximately 10 min.

The questionnaire was structured into three sections (Additional file 1):

- The first section included questions on the socioeconomic and demographic characteristics of the respondents, including age, years of experience, title of academic degree, percentage of workload dispensing to pregnant women out of the total workload, working hours per week, professional status (owner, co-owner, or employee) and engagement in continuous professional development.
- The second section explored the confidence and practices of community pharmacists when dispensing medications during pregnancy, including asking about pregnancy status, switching medications to safer options, assessing eligibility to provide information and dispensing OTC medications.
- The third section explored knowledge on medication safety during pregnancy, where the respondent was provided with four choices (safe; the benefits and risks must be evaluated; not safe; I do not know). The drugs were divided into four categories that listed the most commonly used drugs, in addition to those well known to cause harm to the pregnant woman. The “prescription drugs” category included 25 drugs, such as amoxicillin, azithromycin, enalapril, isotretinoin, and tetracycline. The second category included OTC drugs such as paracetamol, aspirin, and famotidine. The “nutrient” category included vitamins, iron and forms of folic acid. The fourth category included CAMs that are usually available in Palestinian pharmacies, such as castor oil, senna, mint and ginger.

The knowledge score was calculated by adding all correct answers for prescription-only medications and OTC medications, with a range from zero to 40. However, three medications (Enalapril, Ibuprofen, and Bismuth subsalicylate) were excluded from the total scores due to their variable Food and Drug Administration (FDA) pregnancy categories across different trimesters.

### Data analysis

Descriptive and inferential statistics were employed to analyze the data using the 26th version of the Statistical Package for the Social Sciences (SPSS). The mean (± SD) was reported for continuous variables such as age and years of experience. Percentages and frequencies are reported for categorical variables. The Mann‒Whitney U test and Kruskal‒Wallis H test were used to analyze the associations between the knowledge score, a continuous variable, and other categorical variables. Spearman correlation was used to test for correlations between knowledge scores and other continuous variables, including age and years of experience. A *p* value of less than 0.05 was considered to indicate statistical significance.

## Results

### Sociodemographic characteristics

Of the initial 250 questionnaires distributed, 43 were returned or contained missing data. Therefore, a total of 207 questionnaires were included in the data analysis. The mean age of the respondents was 34.1 ± 10.3 years; 130 were female (62.8%), and 77 were male (37.2%) (Table 1). The majority of respondents were married (64.7%), had children (57.0%), and resided in urban areas (62.8%). A significant proportion of the participants were from the Nablus governorate (43.0%). Most of the respondents had graduated from a local university (75.8%) and had only a bachelor’s degree (89.9%), while 16 (7.7%) had a master’s degree and only 5 (2.4%) had a PhD. Regarding employment status, most of the respondents were employees (67.6%), predominantly working in city pharmacies (59.4%) and had participated in continuous professional development programmes (71.0%). The mean professional experience among the respondents was 9.2 ± 9.1 years, with a mean working hours of 46.8 ± 13.9 h per week. Moreover, 32.7% of their total workload involved dispensing drugs to pregnant women.

**Table 1:** Description of the sociodemographic characteristics of the sample

Variable	Frequency (%)
**Sex**	
Male	77 (37.2)
Female	130 (62.8)
**Graduation place**	
Local graduate	157 (75.8)
International graduate	50 (24.2)
**Education degree**	
Bachelor’s	186 (89.9)
Master’s	16 (7.7)
PhD	5 (2.4)
**Educational program participation**	
Yes	60 (29.0)
No	147 (71.0)
**Governate**	
Nablus	89 (43.0)
Qalqilya	36 (17.4)
Salfit	27 (13.0)
Tulkarm	55 (26.6)
**Children**	
Yes	118 (57.0)
No	89 (43.0)
**Marital status**	
Single	73 (35.3)
Married	134 (64.7)
**Pharmacy location**	
City	123 (59.4)
Village	68 (32.9)
Camp	16 (7.7)
**Residency**	
City	130 (62.8)
Village	71 (34.3)
Camp	6 (2.9)
**Employment status**	
Partnership	12 (5.8)
Owner	55 (26.6)
Employee	140 (67.6)
**Total**	**207 (100)**

### Practice and confidence of CPs

Most of the respondents reported that they “always” inquired about pregnancy status (59.9%) and that they had confidence in their knowledge of addressing drug- and health-related concerns specific to pregnant women (72.5%). When faced with uncertainty about drug safety, 82.6% indicated that they would refer to scientific sources, and 48.8% would advise pregnant women to consult their physicians when requesting a potentially contraindicated drug. Nearly half (51.2%) of the participants contacted the prescribing physician to explore safer options. When asked about the safety of OTC medicines during pregnancy, a substantial majority (88.4%) indicated that not all OTC medicines are considered safe. (See Table 2 for more descriptive details of the knowledge about drug and herb safety.)

**Table 2:** Description of the practices and confidence of community pharmacists regarding drug and herb safety during pregnancy

Variable	Frequency (%)
**Do you ask about the state of pregnancy?**	
Always	124 (59.9)
Sometimes	53 (25.6)
Only if expected	29 (14.0)
Never	1 (0.5)
**Do you refer to certain sources if you do not know the safety of a particular drug for pregnant women?**	
No	2 (1.0)
Sometimes	34 (16.4)
Yes	171 (82.6)
**If a pregnant woman wants to buy more of the medicine she is taking, but you know that it is contraindicated during pregnancy. What advice do you give to her?**	
Advise on an alternative medicine which is safer	32 (15.5)
Advice on stopping this medicine immediately	74 (35.7)
Advise to see the doctor again	101 (48.8)
**You have sufficient knowledge to solve the drug and health-related problems of pregnant women**	
Disagree	57 (27.5)
Agree	150 (72.5)
**Do you return to the prescribers to change the prescribed medicine to a safer option?**	
No	9 (4.3)
Sometimes	92 (44.4)
Yes	106 (51.2)
**OTC medicines are safe for pregnant women**	
No	18 (8.7)
Not all medicines	183 (88.4)
Yes	6 (2.9)
Total	207 (100)

The prescribed drugs that were most frequently reported as safe during pregnancy were amoxicillin (98.1%), azithromycin (81.2%), inhaled ipratropium (64.3%) and inhaled budesonide (63.3%), while those most frequently reported as unsafe were tetracycline (88.9%), isotretinoin (85.5%), enalapril (72.5%), ciprofloxacin (72.0%) and oral contraceptives (69.1%) (Table 3). Additionally, mebendazole (49.8%), fluconazole (48.3%), and clarithromycin (45.9%) were the most common drugs identified as requiring further risk-benefit analysis. The most frequently cited safe drugs for OTC medications were folic acid, paracetamol, ferrous gluconate, calcium carbonate and famotidine, while pseudoephedrine and ibuprofen were considered unsafe. In addition, the OTC medications most commonly reported as needing risk-benefit evaluation were dextromethorphan, guaifenesin, caffeine and ibuprofen (Table 4).

**Table 3:** Description of pharmaceutical knowledge about prescription drug safety during pregnancy

Drug	Safe	Must evaluate risk/benefit	Not safe	I don’t know
	Frequency (%)			
Amoxicillin	203 (98.1)	3 (1.4)	1 (0.5)	0 (0.0)
Metformin	120 (58.0)	53 (25.6)	22 (10.6)	12 (5.8)
Azithromycin	168 (81.2)	31 (15.0)	6 (2.9)	2 (1.0)
Clarithromycin	63 (30.4)	95 (45.9)	39 (18.8)	10 (4.8)
Mebendazole	22 (10.6)	103 (49.8)	63 (30.4)	19 (9.2)
Fluconazole	34 (16.4)	100 (48.3)	64 (30.9)	9 (4.3)
Enalapril	4 (1.9)	42 (20.3)	150 (72.5)	11 (5.3)
Amlodipine	27 (13.0)	60 (29.0)	110 (53.1)	10 (4.8)
Bisoprolol	34 (16.4)	78 (37.7)	81 (39.1)	14 (6.8)
Atorvastatin	8 (3.9)	56 (27.1)	129 (62.3)	14 (6.8)
Lamotrigine	19 (9.2)	77 (37.2)	81 (39.1)	30 (14.5)
Phenobarbital	7 (3.4)	52 (25.1)	132 (63.8)	16 (7.7)
Carbamazepine	12 (5.8)	69 (33.3)	105 (50.7)	21 (10.1)
Isotretinoin	2 (1.0)	22 (10.6)	177 (85.5)	6 (2.9)
Metronidazole	73 (35.3)	93 (44.9)	34 (16.4)	7 (3.4)
Acyclovir	27 (13.0)	84 (40.6)	63 (30.4)	33 (15.9)
Inhaled Salbutamol	128 (61.8)	52 (25.1)	17 (8.2)	10 (4.8)
Oral contraceptive	8 (3.9)	40 (19.3)	143 (69.1)	16 (7.7)
Paroxetine	5 (2.4)	57 (27.5)	111 (53.6)	34 (16.4)
Valproic acid	14 (6.8)	52 (25.1)	116 (56.0)	25 (12.1)
Inhaled Ipratropium	133 (64.3)	48 (23.2)	14 (6.8)	12 (5.8)
Inhaled Budesonide	131 (63.3)	62 (30.0)	10 (4.8)	4 (1.9)
Alprazolam	10 (4.8)	40 (19.3)	136 (65.7)	21 (10.1)
Ciprofloxacin	13 (6.3)	36 (17.4)	149 (72.0)	9 (4.3)
Tetracycline	6 (2.9)	12 (5.8)	184 (88.9)	5 (2.4)

**Table 4:** Description of pharmaceutical knowledge about OTC drug safety during pregnancy

Drug	Safe	Must evaluate risk/benefit	Not safe	I don’t know
	Frequency (%)			
Paracetamol	203 (98.1)	4 (1.9)	0 (0.0)	0 (0.0)
Ibuprofen	37 (17.9)	84 (40.6)	85 (41.1)	1 (0.5)
Guaifenesin	82 (39.6)	89 (43.0)	31 (15.0)	5 (2.4)
Pseudoephedrine	30 (14.5)	74 (35.7)	91 (44.0)	12 (5.8)
Aspirin	121 (58.5)	76 (36.7)	8 (3.9)	2 (1.0)
Caffeine	80 (38.6)	89 (43.0)	29 (14.0)	9 (4.3)
Famotidine	184 (88.9)	17 (8.2)	6 (2.9)	0 (0.0)
Dextromethorphan	65 (31.4)	95 (45.9)	39 (18.8)	8 (3.9)
Fluticasone Nasal	101 (48.8)	72 (34.8)	22 (10.6)	12 (5.8)
Loratadine	144 (69.6)	42 (20.3)	13 (6.3)	8 (3.9)
Rectal Lidocaine	60 (29.0)	72 (34.8)	41 (19.8)	34 (16.4)
Bismuth subsalicylate	32 (15.5)	77 (37.2)	59 (28.5)	39 (18.8)
Omeprazole	120 (58.0)	60 (29.0)	25 (12.1)	2 (1.0)
Calcium Carbonate	189 (91.3)	13 (6.3)	2 (1.0)	3 (1.4)
Ferrous gluconate	195 (94.2)	9 (4.3)	0 (0.0)	3 (1.4)
Folic acid	206 (99.5)	1 (0.5)	0 (0.0)	0 (0.0)
Magnesium sulfate	142 (68.6)	43 (20.8)	10 (4.8)	12 (5.8)
Potassium chloride	94 (45.4)	69 (33.3)	13 (6.3)	31 (15.0)

Regarding the use of supplements during pregnancy, vitamin C, vitamin D, vitamin B and zinc acetate were the most frequently considered safe supplements, while vitamin A was reported as unsafe by 65.2% of the respondents (Table 5). Finally, the herbs most frequently considered safe during pregnancy were chamomile, peppermint and anise. In contrast, castor oil, Senna, St. John’s wort and ginseng (42.5%) were the most frequently reported unsafe oils (see Table 6).

**Table 5:** Description of pharmaceutical knowledge about medical supplement safety during pregnancy

Supplement	Safe	Must evaluate risk/benefit	Not safe	I don’t know
	Frequency (%)			
Vitamin A	31 (15.0)	31 (15.0)	135 (65.2)	10 (4.8)
B Vitamins	170 (82.1)	23 (11.1)	6 (2.9)	8 (3.9)
Vitamin C	200 (96.6)	6 (2.9)	1 (0.5)	0 (0.0)
Vitamin D	179 (86.5)	19 (9.2)	4 (1.9)	5 (2.4)
Vitamin E	126 (60.9)	47 (22.7)	19 (9.2)	15 (7.2)
Vitamin K	98 (47.3)	61 (29.5)	30 (14.5)	18 (8.7)
Zinc acetate	156 (75.4)	35 (16.9)	1 (0.5)	15 (7.2)

**Table 6:** Description of pharmaceutical knowledge about medicinal herb safety during pregnancy

Herb	Safe	Must evaluate risk/benefit	Not safe	I don’t know
	Frequency (%)			
Anise	152 (73.4)	30 (14.5)	14 (6.8)	11 (5.3)
Castor oil	17 (8.2)	31 (15.0)	147 (71.0)	12 (5.8)
Chamomile	162 (78.3)	21 (10.1)	12 (5.8)	12 (5.8)
Garlic	151 (72.9)	30 (14.5)	8 (3.9)	18 (8.7)
Ginger	113 (54.6)	50 (24.2)	31 (15.0)	13 (6.3)
Ginseng	27 (13.0)	54 (26.1)	88 (42.5)	38 (18.4)
Parsley	61 (29.5)	56 (27.1)	64 (30.9)	26 (12.6)
Peppermint	158 (76.3)	29 (14.0)	8 (3.9)	12 (5.8)
Psyllium	63 (30.4)	56 (27.1)	46 (22.2)	42 (20.3)
Cinnamon	55 (26.6)	56 (27.1)	79 (38.2)	17 (8.2)
Clove	61 (29.5)	56 (27.1)	51 (24.6)	39 (18.8)
Senna	8 (3.9)	34 (16.4)	143 (69.1)	22 (10.6)
St. John’s Wort	5 (2.4)	39 (18.8)	111 (53.6)	52 (25.1)
Thyme	146 (70.5)	29 (14.0)	21 (10.1)	11 (5.3)

### Knowledge of CPs

The median knowledge score of the participants was 19.00 [16.00–22.00]. A higher knowledge score was significantly associated with receiving a master’s degree (*p* = 0.027) and having participated in a CPD program (*p* = 0.019) (Table 7). Moreover, the knowledge score had a negative but nonsignificant correlation with age (*r*= -0.027, *p* = 0.694) and years of experience (*r* = -0.027, *p* = 0.521) (Table 8).

**Table 7:** Association between the knowledge score and background characteristics of the participants

Variable	Freq. (%)	Median [Q1-Q3]	P value
**Sex**			0.873
Male	77 (37.2)	18.00 [16.00–22.00]	
Female	130 (62.8)	19.00 [16.00-21.25]	
**Graduation place**			0.686
Local graduate	157 (75.8)	19.00 [15.50–22.00]	
International graduate	50 (24.2)	18.50 [16.75-22.00]	
**Education degree**			0.027
Bachelor’s	186 (89.9)	18.00 [16.00–21.00]	
Master’s	16 (7.7)	21.00 [18.25–23.75]	
PhD	5 (2.4)	16.00 [11.50–22.50]	
**Participation in continuous professional development (CPD) programs**			0.019
Yes	60 (29.0)	18.00 [15.00–21.00]	
No	147 (71.0)	19.50 [17.00–22.00]	
**Governorate**			0.063
Nablus	89 (43.0)	20.00 [16.00–22.00]	
Qalqilya	36 (17.4)	18.00 [16.00–20.00]	
Salfit	27 (13.0)	17.00 [15.00–19.00]	
Tulkarm	55 (26.6)	19.00 [16.00–22.00]	
**Children**			0.114
Yes	118 (57.0)	20.00 [16.00–22.00]	
No	89 (43.0)	18.00 [16.00-21.25]	
**Marital status**			0.157
Single	73 (35.3)	20.00 [16.00–22.00]	
Married	134 (64.7)	18.00 [16.00-21.25]	
**Pharmacy location**			0.507
City	123 (59.4)	19.00 [16.00–22.00]	
Village	68 (32.9)	18.00 [16.00-21.75]	
Camp	16 (7.7)	17.00 [16.25–22.50]	
**Residency**			0.722
City	130 (62.8)	19.00 [15.00–22.00]	
Village	71 (34.3)	19.00 [16.00–22.00]	
Camp	6 (2.9)	17.00 [15.50–20.00]	
**Employment status**			0.457
Partnership	12 (5.8)	20.50 [18.00-21.75]	
Owner	55 (26.6)	18.00 [16.00–22.00]	
Employee	140 (67.6)	19.00 [16.00–21.00]	
**Total**	**207 (100)**		

**Table 8:** Correlations between the knowledge score and background characteristics of the participants

		Age	Work experience (years)
Knowledge Score	Correlation Coefficient	-0.027	-0.045
	P value	0.694	0.521

## Discussion

Despite their influence on patients’ drug decisions, pharmacists in Palestine have demonstrated unsatisfactory knowledge about various pharmacological topics, especially in critical conditions such as pregnancy [28–31]. This study aimed to investigate the knowledge and practices of CPs working in Palestine regarding drug use and safety during pregnancy. The study’s findings revealed that, despite some gaps, CPs demonstrate safe practices and adequate knowledge overall. Despite the considerable workload of dispensing drugs to pregnant women, most CPs do not engage in continuous professional development (CPD) to maintain and update their knowledge.

While CPs seem to follow safe practices when dispensing drugs to pregnant women, some responses reflect fair and inadequate practices. For instance, the proportions of CPs who were asked about pregnancy status and those who were referred to physicians to explore safer options were slightly greater than half. This finding might be ascribed to the taboo surrounding discussions of sexual and reproductive matters in public, discouraging open communication of such matters [32]. Especially in a conservative society, asking about reproductive status may be considered an infringement on privacy and a source of embarrassment for women. Even in less conservative societies, such questions may be considered sensitive. For instance, almost half of CPs in a study conducted in Belgium considered “asking about pregnancy status” to be sensitive [33]. Furthermore, routine inquiries about pregnancy status may be unnecessary, as most drugs do not pose a risk to pregnancy.

Knowledge of CPs about the pregnancy safety profile of prescribed and OTC medications seemed adequate, albeit not exceptional. Respondents correctly recognized safe and commonly used medications during pregnancy, such as amoxicillin, paracetamol and famotidine, and highly teratogenic drugs, such as isotretinoin and enalapril. However, only a minority of the respondents reported tetracycline, ciprofloxacin and ibuprofen as medications requiring risk-benefit evaluation, indicating existing gaps in knowledge that should be addressed. The overall adequate knowledge of drug safety during pregnancy obtained by this study contradicts the findings of local studies revealing a lack of knowledge among pharmacists across other pharmacology topics [12, 28–31, 34]. Given that the vulnerability of the fetus and the peculiar pregnancy-related pharmacokinetics necessitate a cautious approach, an extra emphasis is placed on knowledge about drug safety during pregnancy compared to other topics in education and practice. Moreover, pharmacists frequently encounter concerns about pregnancy during their daily practice, enhancing their knowledge in this regard. This is unlike other topics that can be perceived as less serious, such as the use of CAM, or considered more academic than practical for CPs, such as pharmacokinetic parameters.

However, these findings indicate additional gaps in knowledge about herb safety. While correctly identifying castor and Senna oil as unsafe, answers on the safety profile of ginseng (as correctly unsafe) and St. John’s wort and psyllium (as correctly safe) did not constitute the majority. This finding is consistent with an unpublished study conducted among CPs in Palestine, which revealed inadequate knowledge about herb pharmacology, including herb safety during pregnancy [35]. This may be because the safety of herbs is regarded with less emphasis during pharmacy education. However, herbs are commonly used by pregnant women in Palestine, highlighting the importance of improving knowledge about herb pharmacology among CPs [20, 21].

Although global studies have mostly reported unsatisfactory knowledge among pharmacists regarding drug safety during pregnancy, comparison of these studies is challenging due to substantial methodological differences. Most of these studies reported knowledge scores using various assessment tools that address the complexity and relevance of the included questions [7, 33, 36–38]. However, one regional study from Saudi Arabia used a similar survey to assess the knowledge of CPs and revealed comparable results to our study [39]. To address this methodological difficulty, developing a uniform, accurate and validated questionnaire is recommended to measure knowledge of drug safety during pregnancy. This approach would facilitate accurate comparisons between different studies and enhance the internal validity of similar research.

The study findings reveal that CPs in Palestine have a considerable workload related to pregnant women, while CPD programmes designed to respond to this workload are limited. Almost one-third of the workload of CPs involved dispensing drugs to pregnant women, which is higher than that reported in other studies in Qatar and Nepal [37, 38]. The greater access of pregnant women to community pharmacies may be explained by the tendency to seek maternal health services from private providers, leading them to obtain drugs from community pharmacies out of pocket. In contrast, other integrated healthcare systems with universal health coverage usually provide drugs at hospital pharmacies, thus shifting the workload thereto. Indeed, out-of-pocket (OOP) expenditures contribute most to financing healthcare in Palestine [40]. In comparison, OOP expenditures as a percentage of current health expenditures were 39.49% in Palestine and only 9.48% in Qatar in 2020 [41, 42]. Furthermore, most CPs said they did not actively participate in CPD. While pharmacy education in Palestine has recently undergone notable development, opportunities for CPD remain scarce and disorganized. Pharmacists were found to encourage CPD implementation and acknowledge its benefits for their practice [43]. In fact, national guidelines for CPD were published by the Palestinian Ministry of Health in 2017; however, no policies or programs have been implemented yet [44].

A higher knowledge score was significantly associated with having a master’s degree and participating in CPD programs. This emphasizes the importance of lifelong education to maintain pharmaceutical knowledge and improve patient care. Given the rapid advancements in knowledge and technology in healthcare, CPD programs are key to ensuring updated knowledge, maintaining professional standards, and providing safe, effective health services [45–49].

Therefore, a CPD program tailored to enhancing the knowledge of CPs about the safety of drugs during pregnancy is recommended. CPD should encompass academic and practical components and be linked to relicensing. For instance, Lebanon and Jordan have implemented a mandatory CPD scheme where failure to pass a minimal set of credits subjects the pharmacist to temporary suspension of professional registration [50, 51].

### Limitations and strengths

This study has several limitations. First, measurement errors may have affected the findings. Self-reported data on practice may introduce response bias, wherein respondents overestimate their practice level by answering based on idealized expectations instead of actual practice. Moreover, using an invalidated questionnaire might have compromised the measurement of knowledge and practice. Second, the sample was drawn from the northern governorates of the West Bank, limiting generalizability to the Palestinian population of CPs, including pharmacists who studied and trained elsewhere using different curricula. This study was the first in Palestine and among the first in the region to explore the knowledge and practices of CPs regarding drug safety during pregnancy. Furthermore, it simultaneously took place with the continuous development of pharmacy education in Palestine.

## Conclusions

CPs influence patients’ decisions on drug choices during pregnancy. Overall, the findings of this study revealed that CPs have an appropriate level of knowledge and implement safe practices regarding the use and safety of drugs during pregnancy. These findings, however, indicated a lack of knowledge in some domains, particularly in herb pharmacology. Moreover, most CPs did not actively participate in any form of CPD, despite the need to update their knowledge and practices. A master’s degree and participation in a CPD program were associated with higher knowledge scores. Implementing CPD interventions can improve the knowledge and practice of CPs regarding drug and herb safety during pregnancy and other pharmacological topics. Moreover, designing accurate and valid assessment tools to measure knowledge and practice would help enhance the internal validity of future research and facilitate comparisons between studies of different settings.

### Electronic supplementary material

Below is the link to the electronic supplementary material.

Supplementary Material 1

## Data Availability

The datasets used and/or analysed for the current study are available from the corresponding author on reasonable request.

## Abbreviations

CPs: community pharmacists
CPD: continuous professional development
OTC: over-the-counter medications
CAM: complementary and alternative medicines
SPSS: Statistical Package for the Social Sciences program
OOP: out-of-pocket expenditure
