# Supplementary material for: Knowledge and practice of community pharmacists regarding the safety of drugs during pregnancy: a cross-sectional study from a developing country
Source: BMC Pregnancy Childbirth. 2024 Mar 11;24:189. doi: 10.1186/s12884-024-06393-3 (PMC10926555; doi:10.1186/s12884-024-06393-3)
Supplement: Supplementary file 1 — Supplementary Material 1 [file 12884_2024_6393_MOESM1_ESM.doc]

**Additional File 1: Study questionnaires.** This is the final version of the English version that was used to explore the knowledge and practices of community pharmacists (CPs) regarding medications and herb safety during pregnancy.

**English version**

*You are invited to participate in a research study about complementary and alternative medicine. This questionnaire should take about 10 minutes to complete. An interviewer will fill out the questionnaire based on your answer. Participation is voluntary, and responses will be kept anonymous. The results will be used solely for research purposes.*

**Questionnaire**

1. **Demographic characteristics:**

Age:

Sex: (Male, Female)

Years of work in community pharmacy:

University/ Graduation place:

Education degree: (Bachelor’s, Master’s, or PhD)

Are you in continuing education? (Yes, No)

Governate:

Do you have children? (Yes, No)

Marital status: (single, married)

Hours per week:

Dispensing workload to pregnant women (%):

Location of pharmacy: (city, village, or camp)

Residency: (city, village, or camp)

Employment status: ( Partnership, Owner, Employee)

Monthly salary (NIS):

1. **Practice:**

Do you ask about pregnancy state?

1. Always.
2. Sometimes.
3. Only if expected.
4. Never.

Do you return to resources if don’t know?

1. Yes.
2. No.

What are the usual resources you used?

If a pregnant woman wants to buy more of the medicine which she is taking, but you know that it is contraindicated during pregnancy. What advice do you give her?

1. Advise an alternative medicine which is safer.
2. Advise to stop this medicine immediately.
3. Advise to see the doctor again.

I have sufficient knowledge to solve medication and health-related problems of pregnant women:

1. Agree.
2. Disagree.

3. I am confident about giving advice and counseling to pregnant women.

1. Agree.
2. Disagree.

Do you return to the prescribers to change the prescribed medicine to a safer choice?

1. Yes.
2. No.

Do you update of your knowledge regularly?

1. Yes.
2. No.

I am qualified to provide information and prescribe OTC medicines to pregnant women:

1. Strongly agree.
2. Agree.
3. Neutral.
4. Disagree.
5. Strongly disagree.

OTC medicines are safe for pregnant women:

1. Yes.
2. No.
3. Not all medicines.
4. **Pharmaceutical knowledge:**

| **Drug** | **Safe** | **Must evaluate risk/benefit** | **Not safe** | **I don’t know** |
| --- | --- | --- | --- | --- |
|  | | | |
| **Amoxicillin** |  |  |  |  |
| **Metformin** |  |  |  |  |
| **Azithromycin** |  |  |  |  |
| **Clarithromycin** |  |  |  |  |
| **Mebendazole** |  |  |  |  |
| **Fluconazole** |  |  |  |  |
| **Enalapril** |  |  |  |  |
| **Amlodipine** |  |  |  |  |
| **Bisoprolol** |  |  |  |  |
| **Atorvastatin** |  |  |  |  |
| **Lamotrigine** |  |  |  |  |
| **Phenobarbital** |  |  |  |  |
| **Carbamazepine** |  |  |  |  |
| **Isotretinoin** |  |  |  |  |
| **Metronidazole** |  |  |  |  |
| **Acyclovir** |  |  |  |  |
| **Inhaled Salbutamol** |  |  |  |  |
| **Oral contraceptive** |  |  |  |  |
| **Paroxetine** |  |  |  |  |
| **Valproic acid** |  |  |  |  |
| **Inhaled Ipratropium** |  |  |  |  |
| **Inhaled Budesonide** |  |  |  |  |
| **Alprazolam** |  |  |  |  |
| **Ciprofloxacin** |  |  |  |  |
| **Tetracycline** |  |  |  |  |

| **Drug** | **Safe** | **Must evaluate risk/benefit** | **Not safe** | **I don’t know** |
| --- | --- | --- | --- | --- |
|  | | | |
| **Paracetamol** |  |  |  |  |
| **Ibuprofen** |  |  |  |  |
| **Guaifenesin** |  |  |  |  |
| **Pseudoephedrine** |  |  |  |  |
| **Aspirin** |  |  |  |  |
| **Caffeine** |  |  |  |  |
| **Famotidine** |  |  |  |  |
| **Dextromethorphan** |  |  |  |  |
| **Fluticasone Nasal** |  |  |  |  |
| **Loratadine** |  |  |  |  |
| **Rectal Lidocaine** |  |  |  |  |
| **Bismuth subsalicylate** |  |  |  |  |
| **Omeprazole** |  |  |  |  |
| **Calcium Carbonate** |  |  |  |  |
| **Ferrous gluconate** |  |  |  |  |
| **Folic acid** |  |  |  |  |
| **Magnesium sulfate** |  |  |  |  |
| **Potassium chloride** |  |  |  |  |

| **Supplement** | **Safe** | **Must evaluate risk/benefit** | **Not safe** | **I don’t know** |
| --- | --- | --- | --- | --- |
|  | | | |
| **Vitamin A** |  |  |  |  |
| **B Vitamins** |  |  |  |  |
| **Vitamin C** |  |  |  |  |
| **Vitamin D** |  |  |  |  |
| **Vitamin E** |  |  |  |  |
| **Vitamin K** |  |  |  |  |
| **Zinc acetate** |  |  |  |  |

| **Herb** | **Safe** | **Must evaluate risk/benefit** | **Not safe** | **I don’t know** |
| --- | --- | --- | --- | --- |
|  | | | |
| **Anise** |  |  |  |  |
| **Castor oil** |  |  |  |  |
| **Chamomile** |  |  |  |  |
| **Garlic** |  |  |  |  |
| **Ginger** |  |  |  |  |
| **Ginseng** |  |  |  |  |
| **Parsley** |  |  |  |  |
| **Peppermint** |  |  |  |  |
| **Psyllium** |  |  |  |  |
| **Cinnamon** |  |  |  |  |
| **Clove** |  |  |  |  |
| **Senna** |  |  |  |  |
| **St. John’s Wort** |  |  |  |  |
| **Thyme** |  |  |  |  |
